# Impacts of Distribution Data on Accurate Species Modeling: A Case Study of *Litsea auriculata* (Lauraceae)

**DOI:** 10.3390/plants13182581

**Published:** 2024-09-14

**Authors:** Chao Tan, David Kay Ferguson, Yong Yang

**Affiliations:** 1 Co-Innovation Center for Sustainable Forestry in Southern China, College of Life Sciences, Nanjing Forestry University, 159 Longpan Rd., Nanjing 210037, China; tanchao@njfu.edu.cn; 2 Department of Paleontology, University of Vienna, 1090 Vienna, Austria; david.kay.ferguson@univie.ac.at

**Keywords:** Lauraceae, *Litsea auriculata*, MaxEnt, species conservation, species distribution modeling, specimen identification

## Abstract

Global warming has caused many species to become endangered or even extinct. Describing and predicting how species will respond to global warming is one of the hotspots of biodiversity research. Species distribution models predict the potential distribution of species based on species occurrence data. However, the impact of the accuracy of the distribution data on the prediction results is poorly studied. In this study, we used the endemic plant *Litsea auriculata* (Lauraceae) as a case study. By collecting and assembling six different datasets of this species, we used MaxEnt to perform species distribution modeling and then conducted comparative analyses. The results show that, based on our updated complete correct dataset (dataset 1), the suitable distribution of this species is mainly located in the Ta-pieh Mountain, southwestern Hubei and northern Zhejiang, and that mean diurnal temperature range (MDTR) and temperature annual range (TAR) play important roles in shaping the distribution of *Litsea auriculata.* Compared with the correct data, the wrong data leads to a larger and expanded range in the predicted distribution area, whereas the species modeling based on the correct but incomplete data predicts a small and contracted range. We found that only about 23.38% of *Litsea auriculata* is located within nature reserves, so there is a huge conservation gap. Our study emphasized the importance of correct and complete distribution data for accurate prediction of species distribution regions; both incomplete and incorrect data can give misleading prediction results. In addition, our study also revealed the distribution characteristics and conservation gap of *Litsea auriculata*, laying the foundation for the development of reasonable conservation strategies for this species.

## 1. Introduction

With global warming, many species have become fragmented, leading to endanger and even extinction of species [1,2,3]. A large number of species have become adapted to new distribution areas by modifying the pre-existing community composition and ecosystem function [4,5,6]. Understanding the impact of future climate change on species’ suitable habitats is important for the development of species conservation strategies [7,8,9].

**Figure 1:**
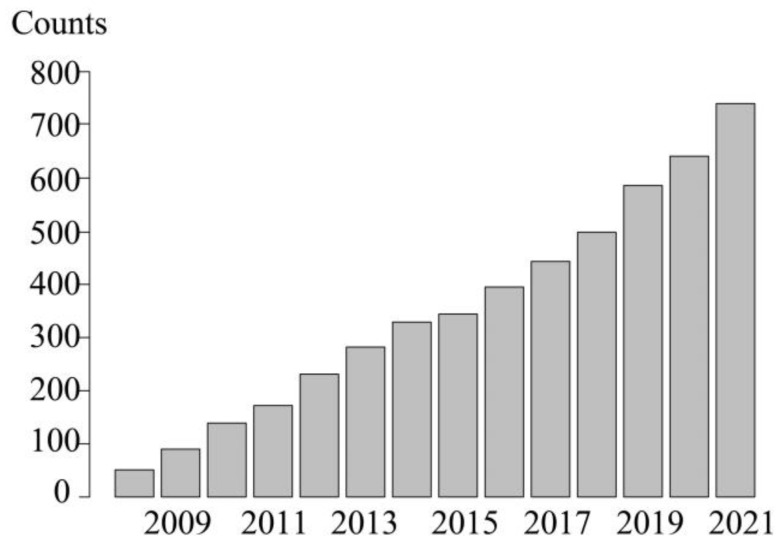
The increasing trend of publications related to species distribution modeling in Web of Science (data extracted using MaxEnt as the keyword: deadline 2022.12).

Species distribution models (SDMs) attempt to associate the distribution information of species with the corresponding environmental variables, establish models, and predict the potential distribution of species in a certain area under specific spatial and temporal conditions in the future, which can quantify the regional and local distribution of species abundance in different scales [10,11]. SDMs mainly include the Generalized Linear Model (GLM), Classification and Regression Tree (CART), Random Forest (RF), Maximum Entropy (MaxEnt), etc. [12,13]. MaxEnt is one of the SDMs based on extant species occurrence records and environmental data. It has the advantage of great accuracy, a small sample size requirement, and good stability, and, thus, has become a widely used modeling approach [14,15,16,17]. In recent years, there has been a steady increase in the literature on SDMs (Figure 1). The widespread use of SDMs has made it possible to predict the potential distribution of species in new space or time [18], and, thus, provides an important reference for species conservation.

SDMs are based on distribution site data and environmental factor data, so uncertainty in the location of species sampling sites will inevitably increase the uncertainty of modeling results [18]. Specimen data have become an important data source for SDM predictions [19]. More than 3000 herbaria in the world have preserved over 400 million plant specimens [20]. With the rapid digitization of plant specimens worldwide, specimen data have widely been used for different purposes, e.g., taxonomy, biogeography, phenology, and SDMs [19,21,22,23]. However, it is worth pointing out that the herbarium collections and digitized specimens contain samples of cultivated plants far from their natural range as well as misidentified materials. There is a lack of quantitative description and research on how these misidentified and non-native specimen data have affected the results of SDMs.

*Litsea auriculata* is a deciduous tree of the family Lauraceae. This species is characterized by scale-like exfoliating bark, large and auriculate leaves, long petioles, black ovoid fruits, and a cup-shaped receptacle. It has important economic and medicinal value; its wood has been used for furniture, while the fruits and roots have been employed as a traditional Chinese medicine (TCM) [24]. *Litsea auriculata* is sporadically distributed in mountainous areas at 500−1500 m in Zhejiang, Anhui, Henan, Hubei etc., and once was listed as an endangered species [25]. As a result, it is important to investigate the conservation status of *Litsea auriculata* and identify any conservation gaps.

Species distribution modeling (SDM) should be based on complete sampling of accurately identified specimens and/or field observation data, which is essential for understanding the suitable distribution area of species and for formulating reasonable conservation strategies. Geng et al. [26] conducted a study on community genetics and ecological niche modeling of *Litsea auriculata* and predicted ecological niche shifts under different climate changes based on data from three populations in the Tianmu Mountain of Zhejiang and the Ta-pieh Mountains of Anhui and Henan. They found that the habitat showed a trend towards contraction and decline in east-central China. However, the sampling range of this study is obviously inadequate, especially for the marginal areas of its distribution range. Based on specimens and literature data, Yang et al. [27] documented the distribution of the species in Zhejiang, Anhui, Henan, Hubei, etc.; Geng et al. [26] did not include these localities. In addition, the Chinese Virtual Herbarium (CVH), the largest digitized herbarium data source, contains misidentified and cultivated specimens. However, it remains unclear how the incomplete sampling and misidentified and cultivated specimen data impact the distribution modeling of this species. SDMs based on the correctly identified and complete distribution data of *Litsea auriculata* are essential for understanding the suitable distribution area of the species and for formulating reasonable conservation strategies.

In this study, we collected and collated six different datasets of *Litsea auriculata* and predicted each dataset using MaxEnt. By doing this, we plan to answer the following questions. (1) What are the impacts of misleading and cultivated specimen data on the results of SDMs? (2) What are the differences between SDM’s results of inadequate sampling and complete and accurate datasets? In addition, we identify the conservation gap of the species based on our new species modeling results and indicate what action needs to be taken to conserve the species.

## 2. Results

### 2.1. Current Distribution Pattern of Litsea auriculata

**Figure 2:**
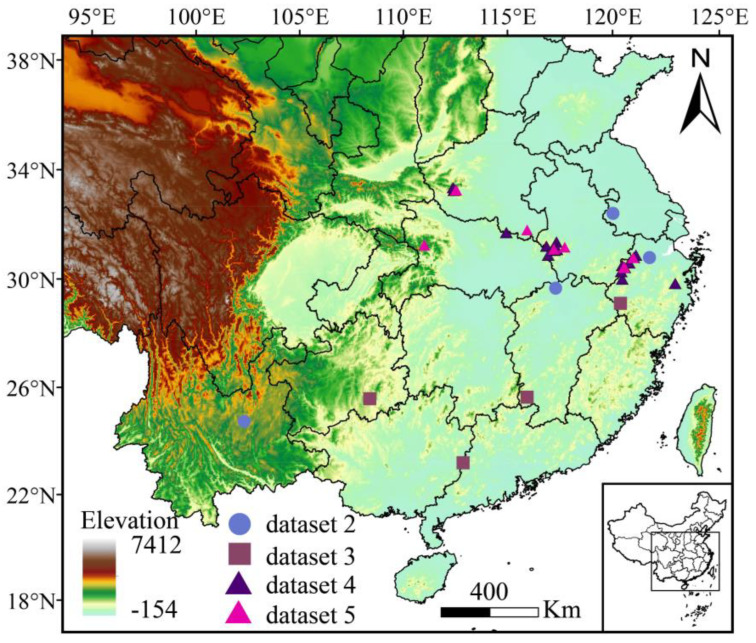
Distribution of *Litsea auriculata* according to different datasets (dataset 1 = dataset 4 ∪ dataset 5; dataset 6 = dataset 1 ∪ dataset 2 ∪ dataset 3).

The prediction result using the correct and complete dataset (dataset 1) showed that *Litsea auriculata* was distributed in Ta-pieh Mountain at the border of Henan and Anhui, Qingliang Peak at the border of Anhui and Zhejiang, and Daming Mountain in Zhejiang. In addition, there was a small number of wild plants distributed in Nanzhao county of Henan and Shennongjia forestry district in Hubei (Figure 2). The species was also cultivated in Xiaoling Mausoleum of Ming Dynasty in Jiangsu, Hangzhou Botanical Garden in Zhejiang, Lushan Botanical Garden in Jiangxi, and Kunming Botanical Garden in Yunnan (Figure 2). Wrong identification records of the specimens expanded the distribution range of the species, including Chongyi in Jiangxi, Fengkai in Guangdong, Jiangshan in Zhejiang, and Sandu Shui Autonomous County of Guizhou (Figure 2).

The six datasets were screened for environmental variables based on Pearson correlation analysis. The results show that dataset 1 and dataset 3 each retained six climate factors in the final MaxEnt model analysis. The remaining datasets retained five climate factors in the final model analysis, respectively (Table 1).

### 2.2. Spatial Pattern and Driving Factors of Potential Distribution Areas of Various Data

Based on the distribution data and environmental data, the potential geographical distribution area of *Litsea auriculata* was simulated using the optimal MaxEnt model. The results show that the AUC value of the simulated curves of all six datasets was greater than 0.994, indicating that the prediction results of the model are very reliable (see Supporting Information Table S2).

**Figure 3:**
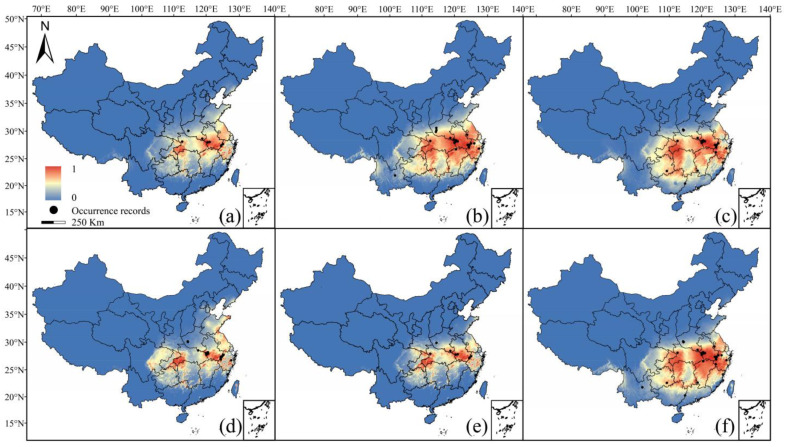
Potential distribution patterns for contemporary climatic conditions of *Litsea auriculata*. (**a**) dataset 1; (**b**) dataset 2; (**c**) dataset 3; (**d**) dataset 4; (**e**) dataset 5; (**f**) dataset 6.

The potential distribution patterns based upon different datasets were significantly different under contemporary climatic conditions. The suitable areas predicted for *Litsea auriculata* based on the correct dataset (dataset 1) were mainly distributed in Ta-pieh Mountain, Huangshan Mountain, southwestern Hubei, and a small area in Zhejiang (Figure 3a). Under-sampled datasets predicted distribution areas showing minor differences from the correct dataset (dataset 1). Compared with the predicted result of dataset 1, the extent of the suitable area based upon the specimen dataset (dataset 4) extended in the easterly and westerly directions and shrunk in the middle part (Figure 3d), while the suitable area based upon the population dataset (dataset 5) shrunk gradually from the periphery to the middle (Figure 3e). The difference between the suitable areas based upon the inaccurate datasets (datasets 2, 3, and 6) and the correct dataset (dataset 1) was rather obvious, and the potential distribution areas based upon these three datasets were widely distributed and extended in all directions, throughout the middle and lower reaches of the Yangtze River (Figure 3b,c,f). Additionally, under the 2050s and 2070s RCP 2.6/4.5/8.5 climate scenarios, the suitable areas based upon these different datasets were basically consistent with those under contemporary conditions (see Supporting Information Figure S1).

**Figure 4:**
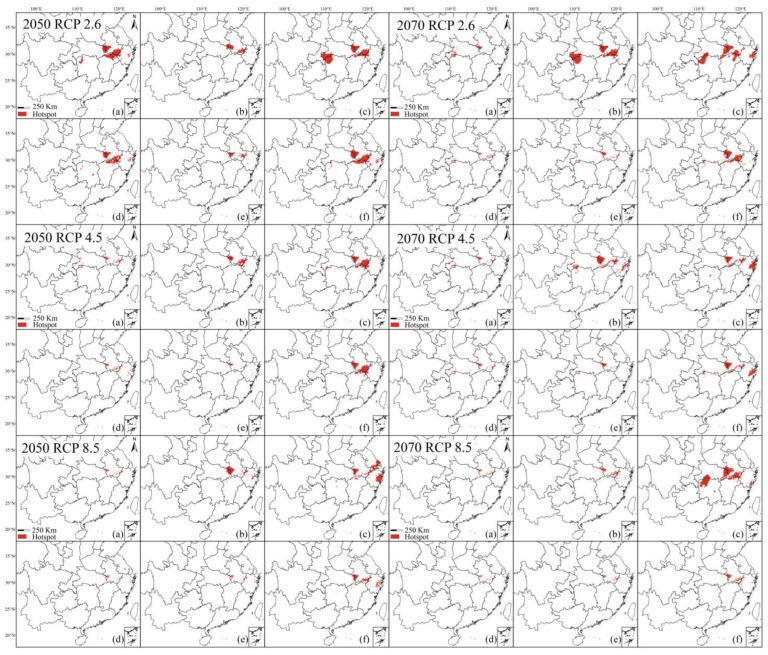
Distribution status of the hotspot regions. (**a**) dataset 1; (**b**) dataset 2; (**c**) dataset 3; (**d**) dataset 4; (**e**) dataset 5; (**f**) dataset 6.

The predicted hotspot areas based upon different datasets showed distinct trends under various climatic conditions (Figure 4). The correct but incomplete datasets (datasets 4 and 5) displayed minor differences from the correct dataset (dataset 1), ranging from 0.01% to 0.54%. The largest hotspot area anomaly was under the 2050s RCP 2.6 condition, where the population dataset (dataset 5) differs from the correct dataset (dataset 1) by 0.54% with an area of 51,900 km^2^. The smallest hotspot area occurred under multiple climate scenarios; the suitable area based on the specimen dataset (dataset 4) differed from that based on the correct dataset (dataset 1) by 0.01% with only 1000 km^2^ under the 2050s RCP 4.5/8.5 climatic conditions. The same result appears in the 2070s RCP 8.5, with the specimen dataset (dataset 4) and population dataset (dataset 5) differing by 1000 km^2^ from the correct dataset (dataset 1).

The incorrect dataset (datasets 2, 3, and 6) and the correct dataset (dataset 1), on the other hand, exhibited a large difference of 0.03–0.88%. The largest hotspot area discrepancy value occurred in the misidentified dataset (dataset 3) for the 2070s RCP 8.5 with 0.88% and an area of 82,600 km^2^. The smallest area gap of 2900 km^2^ occurred in the inclusive dataset (dataset 6) under 2050s RCP 2.6. In addition, the maximum hotspot area occurred in the prediction of the misidentified dataset (dataset 3), except for 2070s RCP 2.6, which occurred in the prediction result of the cultivated dataset (dataset 2) (Table 2).

### 2.3. PCA Analyses of Different Datasets of Litsea auriculata under Different Climatic Conditions

**Figure 5:**
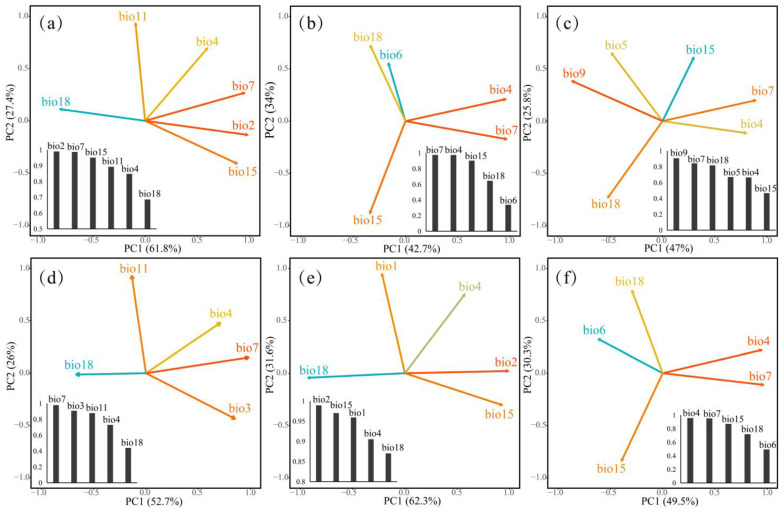
PCA of *Litsea auriculata* in contemporary climatic conditions. (**a**) dataset 1; (**b**) dataset 2; (**c**) dataset 3; (**d**) dataset 4; (**e**) dataset 5; (**f**) dataset 6. The bar chart represents the contribution of variable.

The contribution of environmental variables varied when conducting PCA studies based on different datasets under contemporary climatic conditions. Mean diurnal temperature range (MDTR) and temperature annual range (TAR) played a decisive role in the correct dataset (dataset 1) of *Litsea auriculata* (Figure 5a). TAR and Isothermality (ISN) had the largest impact on the specimen dataset (dataset 4) prediction (Figure 5d), while MDTR and precipitation seasonality (PSN) determined the distribution of the population dataset (dataset 5) (Figure 5e). In the incorrect datasets (datasets 2, 3, and 6), the two most important determinants for the distribution of cultivated (dataset 2) and all recorded datasets (dataset 6) were TAR and temperature seasonality (TSN) (Figure 5b), while the distribution of the misidentified dataset (dataset 3) was limited by mean temperature of the driest quarter (MTDQ) and TAR (Figure 5c).

### 2.4. Distribution and Conservation Status of Litsea auriculata under Various Climatic Conditions Using Different Datasets

**Figure 6:**
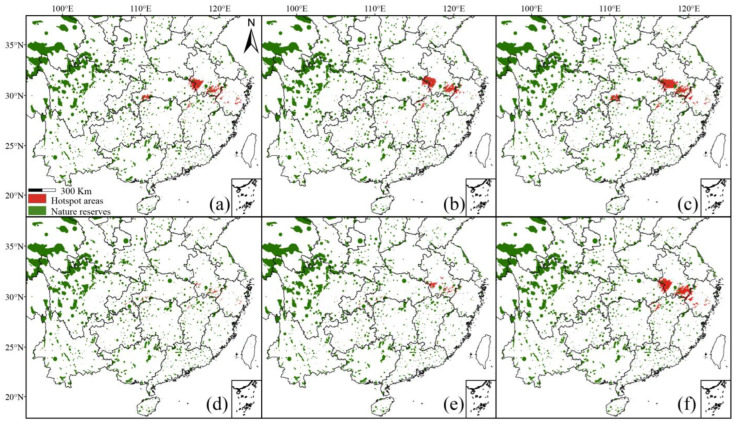
Hotspots located at all altitudes of protected areas under contemporary climate conditions. (**a**) dataset 1; (**b**) dataset 2; (**c**) dataset 3; (**d**) dataset 4; (**e**) dataset 5; (**f**) dataset 6.

Under contemporary climatic conditions, the hotspots and protection status predicted based on the different datasets displayed great discrepancies. The hotspots based on the correct dataset (dataset 1) were mainly distributed in Ta-pieh and Huangshan Mountains, with small stands in southwestern Hubei and Zhejiang. The total area was 15,400 km^2^, of which 3600 km^2^ (23.38%) was located in nature reserves (Figure 6a, Table 3).

The range of the hotspots predicted by the inaccurate datasets (datasets 2, 3, and 6) displayed a certain degree of expansion compared to the correct dataset (dataset 1). The hotspots of the cultivated dataset (dataset 2) are concentrated in the Ta-pieh and Huangshan Mountains, with a small amount of areas in Hunan, with a total area of 28,800 km^2^, of which 2600 km^2^ (9.03%) was in protected areas (Figure 6b, Table 3). The range of hotspots predicted by the inclusive dataset (dataset 6) was similar to that of the cultivated dataset with additional distribution areas in southwestern Zhejiang; the total area of the hotspot range was 36,500 km^2^, with only 3300 km^2^ (9.04%) was located in nature reserves (Figure 6c, Table 3). The misidentified dataset (dataset 3) predicted the largest hotspot area of 40,400 km^2^, which formed a denser area in southwestern Hubei and northwestern Hunan compared with the cultivated dataset, and extended outwards from the Ta-Pieh and the Huangshan Mountains, with only 5300 km^2^ (13.18%) was located in nature reserves (Figure 6f, Table 3).

The distribution range of hotspot regions predicted by the correct but incomplete datasets (datasets 4 and 5) was similar to that predicted by the correct dataset (dataset 1) and showed an overall contraction. The hotspot areas of the population dataset (dataset 4) contracted towards the central areas of the correct dataset (dataset 1) and possessed a total area of 11,500 km^2^ with only 1800 km^2^ (15.65%) in nature reserves (Figure 6e, Table 3). Species modeling based on the specimen dataset (dataset 5) showed a shrinking trend in the hotspots and a scattered occurrence in southwestern Hubei; the total area covered ca. 8600 km^2^ with only 1700 km^2^ (19.77%) of the hotspot area in nature reserves (Figure 6d, Table 3).

## 3. Discussion

### 3.1. Importance of Accurate and Complete Species Distribution Records for Species Distribution Modeling

Accurate and reliable distribution data are the basis for species modeling predictions. Kadmon et al. [28] conducted a comparative study on the distribution modeling of 149 woody plant species in Israel, which revealed that data bias can reduce the accuracy of species modeling. Raes and ter Steege [29] performed a null model test on species modeling and found that modeling with incorrect distribution data showed significantly different results from the correct dataset, demonstrating the impact of data bias on species modeling; the study used a small dataset (less than 20 or 10 records) for modeling, and demonstrated the feasibility of small datasets in species modeling. This was further corroborated by Wolmarans et al. [30] and Chen et al. [31]. In this study, we compared predictions based on datasets containing misidentified records (datasets 1, 2, and 6) with that based on a dataset including correctly identified and complete natural distribution records (dataset 1). We found that the datasets containing misidentified records can result in expansion of the suitable areas, thus significantly reducing the accuracy of the model. We compared the prediction results based on the distribution dataset containing cultivated records with those of the correctly identified and complete natural distribution records, and we found that the suitable distribution area expands greatly from the center to the surrounding area. Our comparative study of species modeling results based on incomplete population distribution records (datasets 4 and 5) and correctly identified, complete population distribution records (dataset 1) suggests that the suitable area showed a conspicuous contraction trend with a very narrow distribution. As a result, species modeling predictions based on such misidentified and inaccurate specimen data can arrive at misleading conclusions.

With the rapid development of digital cameras, computers, and internet and information technology, a large number of specimens in global herbaria have been digitized [19]. As of June 2023, the Global Biodiversity Information Facility (GBIF) harbored 88.4 million specimens. The Australian Biological Atlas (https://www.ala.org.au/, Atlas of Living Australia, ALA) deposits over 6.65 million specimen records. The National Plant Specimen Resource Center (http://www.cvh.ac.cn/, National Plant Specimen Resource Center, NPSRC), the largest digital plant specimen integration platform in China, harbors 8.17 million plant specimen data. There are about 11.46 million digital plant specimens recorded in the National Specimen Information Infrastructure (NSII). These digitized specimens have become important sources for research in biogeography, phenology, and conservation biology, including species distribution model prediction [32,33,34]. However, digitized specimens contain inherent weakness such as misidentified and cultivated records, and are, thus, the main source of erroneous data in species modeling. Incorrect distribution information often leads to severe range deviations and obscures the true species model [35]. As a result, it is necessary to remove and correct the misidentified records and cultivated records before conducting species model predictions.

Because published floras record older data and often contain incomplete information, the integration of floras cannot resolve the problem of data completeness. In this study, we found that the *Flora of China* records the distribution of *Litsea auriculata* in Tianmu Mountain and Tiantai Mountain in Zhejiang and She county in Anhui [24], and misses many other distribution localities. Our new inventory in this study has added the records of *Litsea auriculata* in Hubei and Henan, and Chun’an county in Zhejiang. The *Flora of Anhui* is comprehensive at the county level but remains ambiguous regarding the distribution below the county level. The *Jiangxi Seed Plant List* contains an incorrect record of *Litsea auriculata*, which originates from the misidentified digitized specimens [36]. The distribution information in these botanical catalogs is fragmentary and cannot be used directly for species modeling, and it needs to be verified and integrated. Only when complete and accurate data are available can we obtain valuable research results, which can help understand the distribution characteristics of species and provide important references for biodiversity conservation.

Specimens are the primary source of species distribution data; they should be correctly identified by taxonomists before utilization. Correct identification is fundamental, not only for species distribution modeling, but also for biodiversity conservation. However, taxonomy, as a traditional discipline, is handicapped in the assessment and evaluation system of many different research institutions [37]. Most research funding has been deployed in more fashionable and advanced research fields, e.g., genome sequencing, making it difficult to train traditional taxonomists. As a result, no taxonomists work in the herbaria to correct the misidentifications. To overcome this drawback, it is necessary to promote traditional taxonomy and maintain a permanent taxonomic research team.

### 3.2. Potential Distribution and Conservation Assessment Based on Accurate Identification and Complete Distribution Dataset of Litsea auriculata

In this study, we established a reliable potential distribution area of *Litsea auriculata* based on an accurately identified and complete dataset (dataset 1). The modeling results show that, compared with other plants of the Lauraceae [38], the distribution range of this species is generally northerly and is currently located mainly on mountane forest slopes in the mid-latitudes of central-eastern China. The predicted distribution is similar to the distribution characteristics of gymnosperm species [39,40]. With global warming, in the future, the suitable distribution area of *Litsea auriculata* will tend to contract, and eventually decrease in the central-eastern part of China, as suggested in a previous study [26]. In addition, the predicted hotspot areas under contemporary climate using accurately identified and complete datasets (dataset 1) shifted southwards compared to Geng et al. [26]. This difference may be caused by the bias of distribution data, as Geng et al. [26] did not fully sampled the distribution of the species in the southern regions such as Anhui and Zhejiang.

The potential distribution trend of *Litsea auriculata* shows a clear mismatch with subtropical broadleaf evergreen forest plants. Previous studies have suggested that subtropical broadleaved evergreen forest species will expand northwards and eastwards under future climatic conditions [41,42]. The potential distribution range of *Litsea auriculata* does not vary significantly across time, with an overall range of only 0.09–0.54%, and the only local expansion and contraction occurring in some mountains and plains at the edges of the subtropical broadleaved evergreen forests. Coincidentally, a similar pattern was also found in a study of the genus *Cinnamomum* [43]. In addition, as in many gymnosperms, *Litsea auriculata* may have survived by elevational shifts during the late Quaternary glacial oscillations [44].

The survival of *Litsea auriculata* is at least partially attributable to its habitat dilemma. Previous studies have shown that the genetic structure of *Litsea auriculata* continues to diverge and expand, forming small-scale populations [45]. Increased random genetic variation, high levels of inbreeding, and reduced gene numbers, combined with a progressively warmer climate, have led to a dramatic decline in the distribution area of this species [26]. In our study, the predicted results based on an accurately identified and complete dataset (dataset 1) for hotspot areas of *Litsea auriculata* under contemporary climatic conditions show that the species continues to expand in all directions in the future, with increased fragmentation, a gradual reduction in living space, and a further decrease in area, which is consistent with the results of previous studies. In addition, the narrow and contracted distribution area has increased theendanger of *Litsea auriculata* [46], and irreversible damage may occur if these small areas are disturbed [41].

Species distribution models can suggest the chances of survival of endangered plants and facilitate the development of targeted *in situ* conservation measures [47]. In this study, habitat prediction in combination with the analysis of Chinese nature reserves indicates that only 23.38% of *Litsea auriculata* is currently located in nature reserves, so a large conservation gap remains. The areas outside the nature reserves are mainly located in southern Anhui and west-central and east-central Zhejiang. These areas have suffered from severe deforestation, habitat loss, and habitat fragmentation [48], which may have led to a significant decrease in the number and population size of *Litsea auriculata.* The area of the species within the nature reserve will gradually shrink under the future warming scenario and may even deviate excessively from the reserve in the 2050s RCP 2.6 scenario (Table 4), thus greatly increasing the extinction probability. Therefore, in the face of such a situation, a nature reserve should be established for *Litsea auriculata*, and special staff should be assigned to protect the forest land, prohibit indiscriminate logging practices, and reduce human interference. In accordance with previous studies, we found that a large number of threatened gymnosperms also survive in the distribution area of *Litsea auriculata* [40,49], so it is crucial to strengthen the protection of these areas for other threatened plants as well. In addition, because the genetic differentiation among populations of *Litsea auriculata* is large and gene flow is low [45], it would be beneficial to increase the level of genetic diversity of *Litsea auriculata* if a sufficient number of individuals within all populations could be selected for intensive translocation and conservation.

## 4. Materials and Methods

### 4.1. Data Collection and Processing

#### 4.1.1. Distribution Data of *Litsea auriculata*

The distribution data of *Litsea auriculata* were obtained from the Chinese Virtual Herbarium (CVH), National Specimen Information Infrastructure (NSII) and published literature [24,26,45]. We annotated the data source of each distribution record to generate different datasets. The distribution records were cross-checked. All the specimen records were visually identified by the corresponding author, and misidentified and cultivated records were labeled. Then, the collected data were further processed and separated into six datasets (Table 5): (1) dataset 1 (correct and complete) including all known natural distribution locality from specimens and literature; (2) dataset 2 (cultivated) containing correctly identified and cultivated specimens; (3) dataset 3 (misleading) encompassing correctly identified and misidentified specimens and excluding cultivated specimens; (4) dataset 4 (specimen, correct but incomplete) including only correctly identified specimen data; (5) dataset 5 (population, correct but incomplete) including population localities from literature; (6) dataset 6 (all) containing all the distribution records of populations, specimens (correctly identified, misidentified and cultivated). For occurrence records without geographic coordinates, we used Google Maps (http://maps.google.cn/) to obtain the geographic coordinates of the distribution records. We removed duplicate specimens and redundant records with the different datasets before MaxEnt modeling analysis and imported the distribution data into ArcGIS 10.2 to remove duplicate points, i.e., only one of the distribution points within 10 km was retained [43]. Finally, six distribution datasets were obtained in our study. Dataset 4 contained 16 records and was based on herbarium specimen data from CVH and NSII. Dataset 5 was collected from the literature and contained nine records. Dataset 1 was an integration of datasets 4 and 5 and consisted of a total of 19 records after removing duplicate records. Both datasets 2 and 3 were assembled using specimen data from CVH and NSII, each containing 23 records. Dataset 6 was an integration of dataset 2, dataset 3, dataset 4, and dataset 5, and contained a total of 27 records after deleting duplicate records.

#### 4.1.2. Environment Variable Data

Altogether, 19 Environment variables data at 2.5′ resolution were downloaded from WorldClim v 2.1 (https://www.worldclim.org/) (see Table S1), including current climatic data (1970–2000) and future climate predictions. The future climatic data were based on the climate model of the Beijing Climate Center Climate System Model version 1.1 (BCC-CSM 1.1), which was constructed under RCP 2.6, RCP 4.5, and RCP 8.5 for 2050 (average value over the period 2041–2060) and 2070 (average value over the period 2061–2080) for the three representative concentration pathways (RCPs) [50].

The climate data layers were extracted using ArcGIS 10.2, and the extracted layers were converted to ASCII format. In order to avoid influencing the final assessment of the model of high correlations between environmental variables [51], Pearson correlation analysis was performed on 19 climatic variables for each period using the cor function of R 4.4.1 (https://www.r-project.org/), and the climatic factors in r < |0.85| that were more closely related to species distribution were retained [52,53]. Finally, we performed principal component analysis (PCA) on the variables under contemporary climatic conditions to identify the key drivers influencing the distribution of *Litsea auriculata.*

### 4.2. Potential Distribution Prediction Based on MaxEnt Model

Firstly, we imported the six distribution data types (.CSV format) and climatic data (.ASC format) for each period into MaxEnt 3.4.1 software for species ecological niche simulation. Secondly, different procedures for simulating the potential distribution were performed for datasets with different sample sizes. For datasets with less than 25 coordinate points, the Jackknife method was used for simulation evaluation. For species modeling, one of the coordinates was removed and the model was established based on the remaining n − 1 coordinates, so that n models could be established, and the optimal model was selected for the MaxEnt ecological niche simulation. For datasets with more than 25 available coordinate points, 75% of the species distribution data was set as the training set and 25% as the test set, the number of operational iterations was set to 10, and the rest was used as default values [43,54]. The area under curves (AUC) with receiver operator characteristic (ROC) was used to evaluate the reliability of the simulation results [55]. The range of AUC values is 0 to 1, those closer to 1 indicating the higher reliability of the simulation. The simulation result was considered to be very accurate when the AUC value was between 0.9 and 1, accurate when the AUC was 0.8–0.9, average when the AUC was between 0.7 and 0.8, and unreliable when the AUC result was less than 0.7 [56,57]. Finally, the simulation results of MaxEnt were entered into ArcGIS 10.2 software and transformed into raster layers for visualization, and the natural breaks method was selected to calculate the fitness index P. Based on previous studies, *p* > 0.75 was used as a hotspot for species survival [58], and the proportion of the area in different distribution data types was calculated.

### 4.3. Calculating Hotspots in Protected Areas

To describe and evaluate the local conservation status of *Litsea auriculata*, we assembled 2569 nature reserves (including 440 national nature reserves and 2129 subnational nature reserves) established during 1956–2021 [59]. In ArcGIS 10.2, the base map data of China’s nature reserves superimposed on the samples were used to calculate the area of the contemporary hotspot area located within the reserve, and to evaluate the protection efficiency of *Litsea auriculata*.

## 5. Conclusions

It remains ambiguous how the identification errors, cultivated collections, and data incompleteness impact species distribution modeling. We assembled six datasets and made a comparative study here. We show that misidentification, cultivated specimen data, and data incompleteness all have significant effects on species modeling prediction results. In order to achieve an appropriate prediction of species modeling, one should (1) pay attention to the diversity of collected data to complement each other and improve the completeness of the data set, and (2) screen and verify the data, especially the digitized specimen data, and review them item by item to correct misidentified records and remove cultivated specimen records to enhance the correctness of the data. We assembled the most complete distribution data of *Litsea auriculata* and identified new areas of potential distribution of the species, revealing that the current main distribution range of *Litsea auriculata* is located in the mountane areas of the middle and lower reaches of the Yangtze River, with a tendency to contract in future climatic change scenarios. In addition, our assessment of the conservation status of *Litsea auriculata* reveals that, currently, about 23.38% of the suitable areas for the species have been protected in nature reserves, so there are still relatively large conservation gaps. The resulting information can be used to support management, conservation, and recovery plans for *Litsea auriculata*.

## Figures and Tables

**Table 1:** The environtmental variables of different datasets determined by Pearson correlation analysis and applied to the final MaxEnt model analysis. Detailed information of each climate variables (e.g., bio1,2,3, etc.) see Table S2. (‘*’ indicates the environmental variables used for modeling).

Type	bio1	2	3	4	5	6	7	8	9	10	11	12	13	14	15	16	17	18	19
dataset 1		*		*			*				*				*			*	
dataset 2				*		*	*								*			*	
dataset 3				*	*		*		*						*			*	
dataset 4			*	*			*				*							*	
dataset 5	*	*		*											*			*	
dataset 6				*		*	*								*			*	

**Table 2:** The proportion of hotspot areas in different datasets (hotspots/selected regions).

Type	Present	2050s			2070s		
		RCP2.6	RCP4.5	RCP8.5	RCP2.6	RCP4.5	RCP8.5
dataset 1	0.16%	0.70%	0.14%	0.07%	0.39%	0.15%	0.08%
dataset 2	0.30%	0.29%	0.30%	0.31%	0.92%	0.25%	0.18%
dataset 3	0.42%	0.92%	0.50%	0.57%	0.91%	0.46%	0.96%
dataset 4	0.09%	0.48%	0.13%	0.08%	0.12%	0.12%	0.07%
dataset 5	0.11%	0.16%	0.07%	0.10%	0.11%	0.12%	0.07%
dataset 6	0.38%	0.67%	0.47%	0.30%	0.53%	0.43%	0.19%

**Table 3:** The hotspots areas and the area and proportion of the hotspots in nature reserves according to species modeling using different datasets under contemporary climatic conditions (unit: square kilometers).

Type	Hotspot Areas (km^2^)	Nature Reserves Areas (km^2^)	Proportion
dataset 1	15,400	3600	23.38%
dataset 2	28,800	2600	9.03%
dataset 3	40,400	5300	13.18%
dataset 4	11,500	1800	15.65%
dataset 5	8600	1700	19.77%
dataset 6	36,500	3300	9.04%

**Table 4:** Predicted hotspot area of *Litsea auriculata* based on correct and complete dataset (dataset 1) and area located within nature reserves.

Period	Hotspot Areas	Nature Reserves Areas	Proportion
Present	1.54	0.17	23.38%
2050s RCP2.6	6.73	0.64	9.06%
2050s RCP4.5	1.35	0.22	16.30%
2050s RCP8.5	0.67	0.13	19.40%
2070s RCP2.6	3.75	0.29	7.73%
2070s RCP4.5	1.44	0.23	15.97%
2070s RCP8.5	0.77	0.12	15.58%

**Table 5:** Comparison of six datasets in this study.

Dataset	Features	Sources	Numbers
dataset1	correct	specimens, literature	18
dataset2	cultivated	specimens, literature	22
dataset3	misleading	specimens, literature	22
dataset4	specimen	specimens	16
dataset5	population	literature	9
dataset6	all	specimens, literature	26

## Data Availability

All data used in the study are included in this paper are available in the supporting datasets.

## Supplementary Materials

The following supporting information can be downloaded at: https://www.mdpi.com/article/10.3390/plants13182581/s1, Figure S1: Potential distribution patterns for different climate scenarios conditions except contemporary climatic conditions; Table S1: Six sets of distribution data for this study; Table S2: Climatic variables in the Pearson correlations analysis; Table S3: Contribution rates and AUC values of variables in each period under the MaxEnt model; Table S4: Cumulative variability of PCA analysis.
