# Supplementary material for: Impacts of Distribution Data on Accurate Species Modeling: A Case Study of Litsea auriculata (Lauraceae)
Source: Plants (Basel). 2024 Sep 14;13(18):2581. doi: 10.3390/plants13182581 (PMC11435344; doi:10.3390/plants13182581)
Supplement: Supplementary file 1 [file plants-13-02581-s001.zip › plants-3139062-supplementary.pdf]

Supplementary Materials.

Figure S1. Potential distribution patterns for different climate scenarios conditions except contemporary climatic conditions.

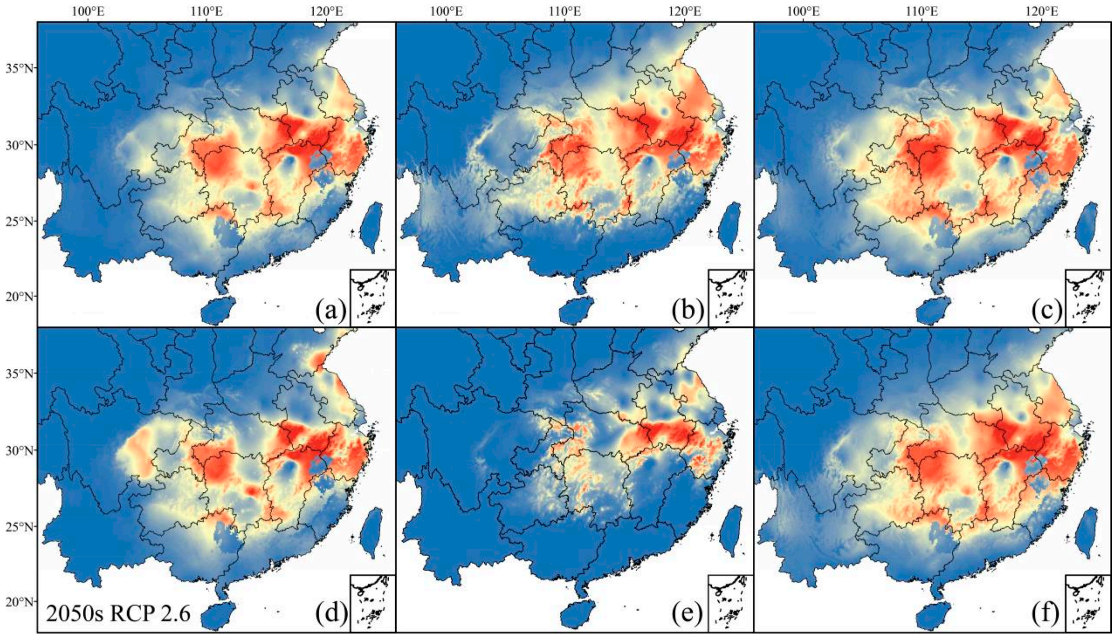

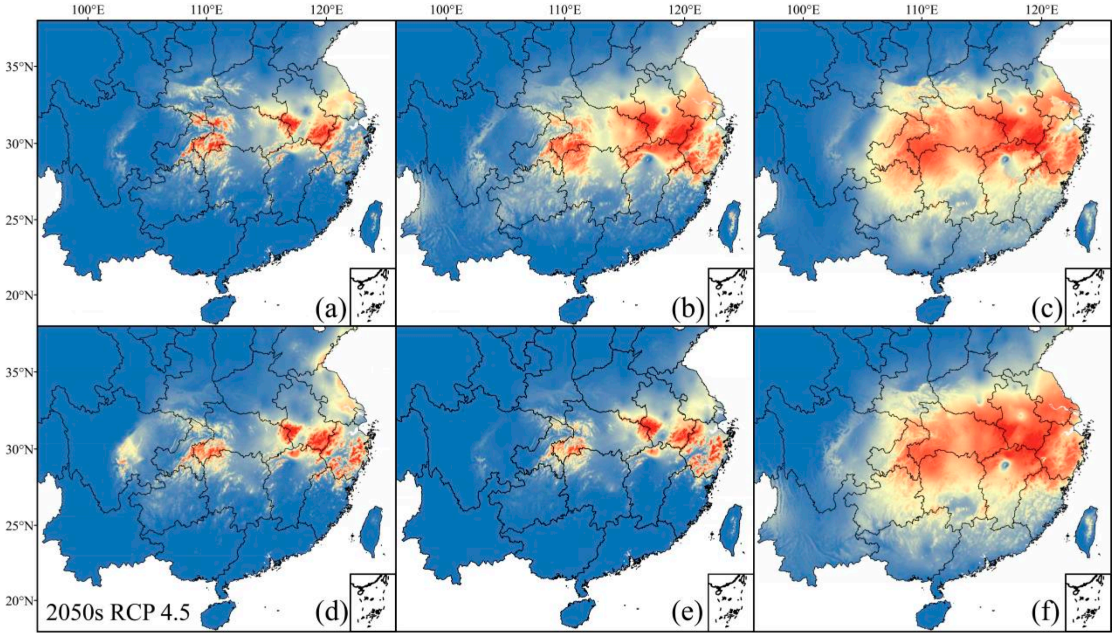

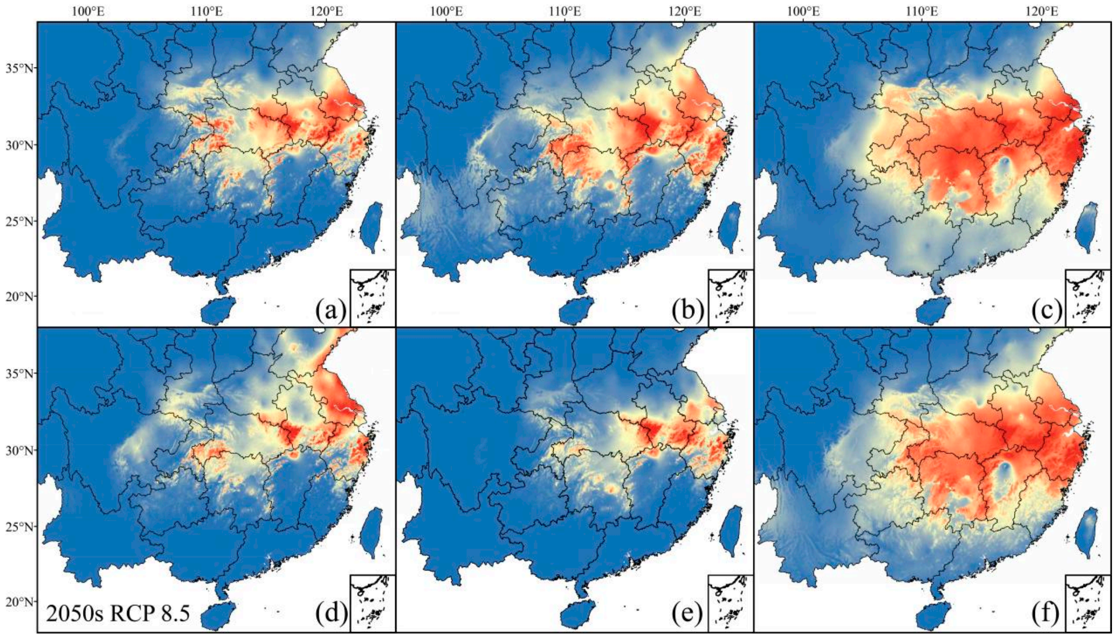
100°E
110°E
120°E
35°N
30°N
25°N
20°N
(a)
(b)
(c)
(d)
(e)
(f)
2050s RCP 8.5

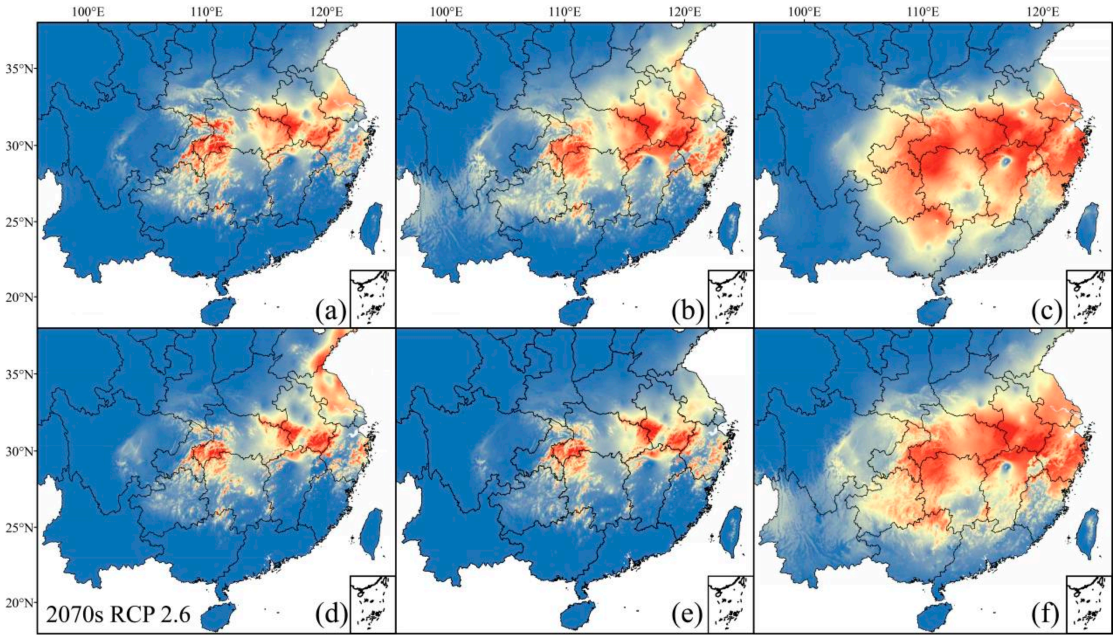
100°E
110°E
120°E
35°N
30°N
25°N
20°N
(a)
(b)
(c)
(d)
(e)
(f)
2070s RCP 2.6

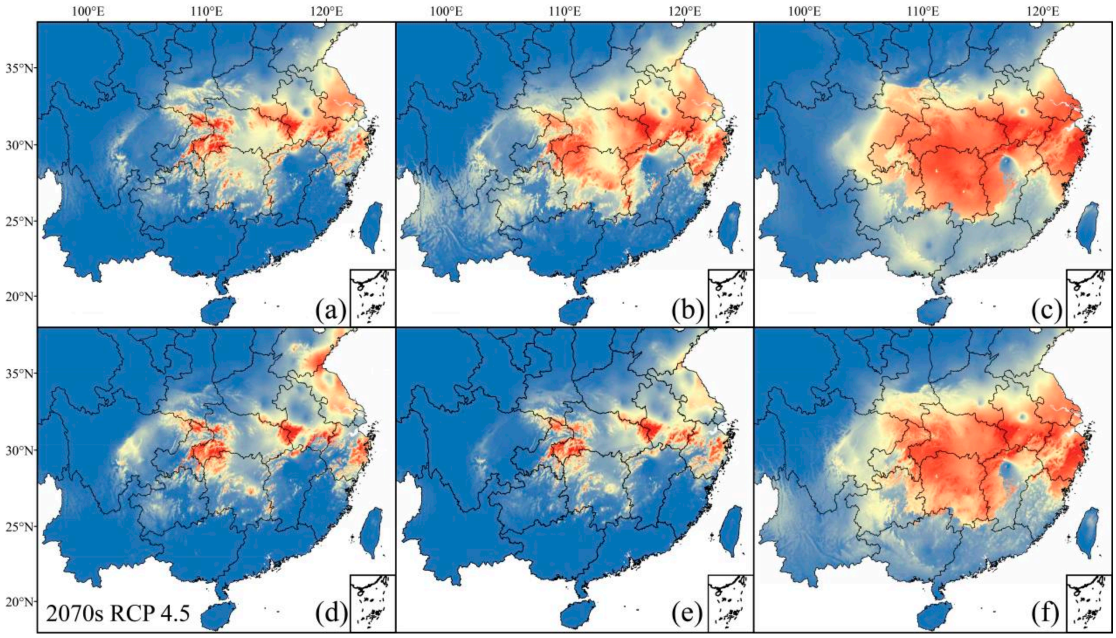

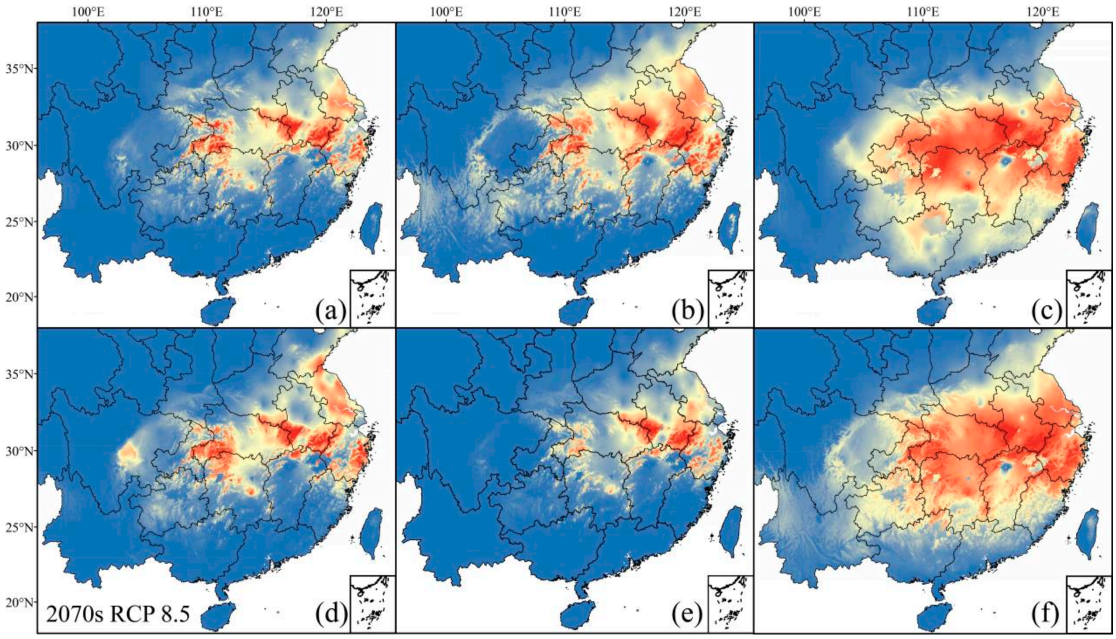

Table S1. Six sets of distribution data for this study.

Num.	Lon	Lat	Location
1	119.43045	30.33988	Tianmu Mountain, Lin'an Conuty, Hangzhou City, Zhejiang Province.
2	119.0015	30.04316	Qingliang Mountain, Lin'an Conuty, Hangzhou City, Zhejiang Province.
3	118.97306	30.03688	Daming Mountain, Lin'an Conuty, Hangzhou City, Zhejiang Province.
4	116.54883	31.05646	Shucheng Conuty, Anqing City, Anhui Province.
5	116.06971	31.0315	Yuexi Conuty, Anqing City, Anhui Province.
6	114.99985	31.8417	Jigong Mountain, Xinyang City, Anhui Province.
7	112.01433	33.53566	Nanzhao Conuty, Nanyang City, Anhui Province.
8	112.0007	33.51875	Nanzhao Conuty, Nanyang City, Anhui Province.
9	110.45608	31.57976	Shennongjia forestry district, Hubei Province.

10	111.94913	33.47106	Baotianman, Neixiang Conuty, Nanyang City, Anhui Province.
11	111.88049	33.62065	Song Conuty, Luoyang City, Anhui Province.
12	115.76637	31.16413	Baimazhai, Jinzhai City, Lu'an City, Anhui Province.
13	116.21261	31.2774	Huoshan Conuty, Lu'an City, Anhui Province.
14	118.63066	30.14395	Jixi Conuty, Xuancheng City, Anhui Province.
15	118.83808	29.88974	She Conuty, Huangshan City, Anhui Province.
16	118.83641	29.61234	Chun'an Conuty, Hangzhou City, Zhejiang Province.
17	121.02821	29.19197	Tiantai Mountain, Tiantai Conuty, Tiantai City, Zhejiang Province.
18	115.79836	30.82973	Yingshan City, Hubei Province.
19	118.84008	32.06338	Ming Xiaoling Mausoleum, Nangjing City, Jiangsu Province.
20	120.12551	30.26051	Hangzhou Botanical Garden, Hangzhou City, Zhejiang Province.
21	115.96899	29.5986	Jiujiang City, Jiangxi Province.
22	102.74918	25.14646	Kunming Botanical Garden, Kunming City, Jiangxi Province.
23	114.30536	25.67648	Congyi County, Ganzhou City, Jiangxi Province.
24	111.51968	23.41995	Fengkai County, Zhaoqing City, Jiangxi Province.
25	118.61116	28.73906	Jiangshan County, Qujiang City, Zhejiang Province.
26	107.86838	25.98558	Sandu Shui Autonomous County, Yunnan Province.
27	119.455097	30.340834	Tianmu Mountain, Lin'an Conuty, Hangzhou City, Zhejiang Province.
28	118.83641	29.61234	Chunan County, Hangzhou City, Zhejiang Province.
29	118.913359	30.114319	Qingliang Mountain, Lin'an Conuty, Hangzhou City, Zhejiang Province.
30	119.014479	30.032257	Daming Mountain, Lin'an Conuty, Hangzhou City, Zhejiang Province.
31	119.224435	30.168569	Chnaghua vallege, Lin'an County, Hangzhou City, Zhejiang Province.
32	119.567251	30.412984	Taihuyuan, Lin'an County, Hangzhou City, Zhejiang Province.
33	121.04204	29.162747	Tiantai Mountain, Tiantai Conuty, Tiantai City, Zhejiang Province.
34	118.838081	29.889742	She Conuty, Huangshan City, Anhui Province.
35	116.21261	31.2774	Huoshan Conuty, Lu'an City, Anhui Province.
36	116.085813	30.988115	Yuexi Conuty, Anqing City, Anhui Province.
37	116.201703	31.002492	Qingtian Vallage, Yuexi Conuty, Anqing City, Anhui Province.
38	115.766373	31.164134	Baimazhai, Jinzhai City, Lu'an City, Anhui Province.
39	114.083414	31.81925	Jigong Mountain, Xinyang City, Anhui Province.
40	111.919478	33.51763	Baotianman, Neixiang Conuty, Nanyang City, Anhui Province.
41	111.880495	33.620659	Shangzhuangping, Song County, Henan Province.
42	115.79836	30.82973	Yingshan City, Hubei Province.

Note：1-18 (dataset 1); 1-22 (dataset 2); 1-18, 23-26 (dataset 3); 27-42 (dataset 4); 1-9 (dataset5); 1-26(dataset 6)

Table S2. Climatic variables in the Pearson correlations analysis.

Climatic variable
BIO1 = Annual Mean Temperature
BIO2 = Mean Diurnal Range (Mean of monthly (max temp - min temp))
BIO3 = Isothermality (BIO2/BIO7) (×100)
BIO4 = Temperature Seasonality (standard deviation ×100)
BIO5 = Max Temperature of Warmest Month

BIO6 = Min Temperature of Coldest Month
BIO7 = Temperature Annual Range (BIO5-BIO6)
BIO8 = Mean Temperature of Wettest Quarter
BIO9 = Mean Temperature of Driest Quarter
BIO10 = Mean Temperature of Warmest Quarter
BIO11 = Mean Temperature of Coldest Quarter
BIO12 = Annual Precipitation
BIO13 = Precipitation of Wettest Month
BIO14 = Precipitation of Driest Month
BIO15 = Precipitation Seasonality (Coefficient of Variation)
BIO16 = Precipitation of Wettest Quarter
BIO17 = Precipitation of Driest Quarter
BIO18 = Precipitation of Warmest Quarter
BIO19 = Precipitation of Coldest Quarter

Table S3. Contribution rates and AUC values of variables in each period under the MaxEnt model.

Period	Correct (dataset 1)			Cultivated (dataset 2)		
	variables	Contribution	AUC	variables	Contribution	AUC
Present	Bio18	61.7%	0.997	Bio18	61.8%	0.997
	Bio4	25.2%		Bio4	23.3%	
	Bio11	8.8%		Bio6	7.8%	
2050	Bio18	64.5%	0.997	Bio18	56.2%	0.996
Rcp2.6	Bio4	27%		Bio4	24.1%	
	Bio15	5.3%		Bio6	15.1%	
2050	Bio18	55.7%	0.998	Bio18	54.8%	0.997
Rcp4.5	Bio4	21.3%		Bio4	23.1%	
	Bio11	13.9%		Bio6	17.5%	
2050	Bio18	51%	0.998	Bio18	54.2%	0.997
Rcp8.5	Bio4	21.9%		Bio4	24.3%	
	Bio11	15.4%		Bio6	19.4%	
2070	Bio11	57.6%	0.998	Bio18	60%	0.996
Rcp2.6	Bio18	23.4%		Bio4	24%	
	Bio2	11.7%		Bio6	13.9%	
2070	Bio18	61.9%	0.998	Bio18	55.7%	0.996
Rcp4.5	Bio4	22.8%		Bio6	22.8%	
	Bio11	11.4%		Bio4	18.6%	
2070	Bio18	62.7%	0.997	Bio18	58.9%	0.997
Rcp8.5	Bio4	22.8%		Bio4	19.8%	
	Bio11	8.3%		Bio6	19.6%	
Period	Specimen (dataset 4)			Misleading (dataset 3)		
	variables	Contributio	AUC	variables	Contribution	AUC

		n				
Present	Bio18	63.9%	0.997	Bio18	64.6%	0.996
	Bio4	23.8%		Bio4	24.8%	
	Bio11	7.2%		Bio15	5.5%	
2050	Bio18	65.8%	0.997	Bio18	61.5%	0.995
Rcp2.6	Bio4	28.5%		Bio4	25.8%	
	Bio7	5%		Bio15	7.3%	
2050	Bio18	61.5%	0.997	Bio18	57.8%	0.996
Rcp4.5	Bio4	25.3%		Bio4	23.8%	
	Bio11	7.4%		Bio15	10.2%	
2050	Bio18	54.7%	0.997	Bio18	54.8%	0.995
Rcp8.5	Bio4	26.3%		Bio4	25.6%	
	Bio11	12%		Bio15	9%	
2070	Bio18	62.2%	0.997	Bio18	62.5%	0.996
Rcp2.6	Bio4	23.8%		Bio4	26.6%	
	Bio11	9.4%		Bio15	6.2%	
2070	Bio18	63%	0.997	Bio18	58.9%	0.995
Rcp4.5	Bio4	24.3%		Bio4	25.2%	
	Bio11	7.4%		Bio15	8.2%	
2070	Bio18	64.9%	0.997	Bio18	63.4%	0.996
Rcp8.5	Bio4	25.3%		Bio4	25.3%	
	Bio7	5.2%		Bio7	6.8%	

Period	Population (dataset 5)			All (dataset 6)		
	variables	Contribution	AUC	variables	Contribution	AUC
Present	Bio15	58.8%	0.998	Bio18	64.6%	0.996
	Bio2	28.7%		Bio4	24.5%	
	Bio18	5.2%		Bio6	4%	
2050	Bio18	48.7%	0.998	Bio18	59.7%	0.995
Rcp2.6	Bio2	27.3%		Bio4	23.9%	
	Bio4	11.3%		Bio6	9.8%	
2050	Bio15	50.6%	0.998	Bio18	57%	0.995
Rcp4.5	Bio18	21.3%		Bio4	24.8%	
	Bio2	10.9%		Bio6	12.5%	
2050	Bio15	76.4%	0.999	Bio18	48.4%	0.995
Rcp8.5	Bio2	9.3%		Bio6	22.7%	
	Bio18	5.6%		Bio4	17%	
2070	Bio2	33.8%	0.998	Bio18	61.3%	0.995
Rcp2.6	Bio15	27.4%		Bio4	23.3%	
	Bio18	8.1%		Bio6	11.1%	
2070	Bio15	46.1%	0.998	Bio18	57.8%	0.994
Rcp4.5	Bio2	28.7%		Bio4	21.7%	
	Bio18	12.1%		Bio6	12.2%	
2070	Bio18	68.9%	0.997	Bio18	63.1%	0.994

Rcp8.5	Bio2	13.4%	Bio4	22.9%
	Bio4	10.6%	Bio6	11.2%

Table S4. Cumulative variability of PCA analysis.

Type	PC1	PC2	PC3
dataset 1	61.82%	89.25%	
dataset 2	42.71%	76.74%	95.51%
dataset 3	47.02%	72.83%	94.07%
dataset 4	41.5%	81.1%	
dataset 5	52.74%	78.72%	95.89%
dataset 6	62.26%	93.84%	
